# Toxic Effect of Silver and Platinum Nanoparticles Toward the Freshwater Microalga *Pseudokirchneriella subcapitata*

**DOI:** 10.1007/s00128-015-1505-9

**Published:** 2015-03-06

**Authors:** Małgorzata Książyk, Monika Asztemborska, Romuald Stęborowski, Grażyna Bystrzejewska-Piotrowska

**Affiliations:** Isotope Laboratory, Faculty of Biology, University of Warsaw, Miecznikowa 1, 02-096 Warsaw, Poland

**Keywords:** Silver nanoparticles, Platinum nanoparticles, Microalga, *Pseudokirchneriella subcapitata*, Toxicity test

## Abstract

The growing use of nanoparticles in a wide range of products has resulted in their release into the aquatic environment; therefore, an understanding of the toxic effects of nanoparticles on aquatic organisms is of permanent importance. The aim of this study was to evaluate the toxicity of silver and platinum nanoparticles toward the freshwater microalga, *Pseudokirchneriella subcapitata*. Algal growth and photosynthetic pigments were determined to quantitate the effects of varying concentrations of Ag and Pt nanoparticles. The silver nanoparticles were much more toxic than the platinum ones. The concentrations causing total inhibition of algal growth were 5.0 and 22.2 mg L^−1^, respectively. Similar results were obtained by analyzing the concentration of photosynthetic pigments in *P. subcapitata* exposed to nanoparticles. Thus, simple spectrophotometric determination of chlorophyll is a convenient tool for the analysis of nanoparticle toxicity to algae.

Nanotechnology is one of the most rapidly expanding fields of technology and, consequently, new nanoproducts are entering more and more areas of everyday life (Bystrzejewska-Piotrowska et al. 2009). An byproduct of this is an increasing risk of environmental exposure to nanotechnology-based materials. Nanoparticles are defined as particles in which at least one of the dimensions does not exceed 100 nm. They exhibit unique physicochemical (e.g., magnetic, optical and electrochemical) properties and can lead to unexpected health or environmental hazards.

Silver nanoparticles (Ag NPs) are the largest and fastest growing class of metal-NPs in product applications (Ahamed et al. 2010). They exhibit high electrical and thermal conductivity, scattering, chemical stability, catalytic activity and non-linear optical behavior; however, it is the exceptional broad-spectrum bactericidal activity of silver and the relatively low cost of manufacturing Ag NP (Capek 2004) that has made them extremely popular in a broad range of consumer materials, including plastics, soaps, pastes, metals and textiles (Frattini et al. 2005). Nanoparticles of platinum (Pt NPs) have attracted attention for industrial applications owing to their remarkable catalytic properties. The effectiveness of catalytic processes on nanoparticles is much higher in comparison with micro-sized particles, because of the expanded reactive surface. Platinum nanoparticles are used in automotive exhaust converters and biomedical applications (Bhattacharya and Murkherjee 2008), or as electrochemical sensors and biosensors (Luo et al. 2006).

Paralleling the use of NPs in a wide range of goods, they are released to the environment, including aquatic systems; however, data on their fate and behavior are scare and the effects of nanoparticles on aquatic organisms remain to be evaluated.

Investigations into the toxicity of Ag NPs have already been undertaken. The action of Ag NPs on diverse aquatic vertebrates, invertebrates, algae and bacteria has been summarized by Fabrega et al. (2011). It has been concluded that concentrations of Ag NPs as low as just a few ng/L can affect prokaryotes, invertebrates and fish. The toxicity of Pt NPs has been investigated for human cells (Elder et al. 2007), but only a few studies considering the effects of nanosized platinum on aquatic organisms have been published so far (Asharani et al. 2011).

The aim of this study was to examine the toxic effects of silver and platinum nanoparticles on the freshwater microalga *Pseudokirchneriella subcapitata* using the algal growth inhibition test. *P. subcapitata* (also known as *Selenastrum capricornutum* or *Rhapidocelis subcapitata*) is very sensitive to heavy metals (Blinova 2004; Kahru et al. 2005) and is a model freshwater alga in toxicology studies. Additionally, the content of chlorophyll was determined as an indicator of the performance of the algae. To date, no studies have reported on the toxicity of non-coated silver nanoparticles or platinum nanoparticles toward *P. subcapitata*.

## Materials and Methods

Nanoparticles of platinum [Pt NPs, nanopowder, particle size <50 nm (TEM), spec. surface area BET 98 m^2^/g] and silver (Ag NPs, nanopowder, particle size <100 nm) were purchased from Sigma-Aldrich, Poland. The particle size and morphology were assessed using a LEO 912AB scanning electron microscope (Carl Zeiss Merlin, USA); the analysis was performed using 1 mmol L^−1^ water suspensions of Ag and Pt NPs.

The commercially available Algaltoxkit FTM (Creasel, Belgium) was used. In the test, the effect of various concentrations of the NPs on the growth rate of the alga *P. subcapitata* was measured. The initial algal culture was prepared from immobilized algal beads, pre-grown in a sterile growth medium as described in the instructions. The initial density of cells was 10^6^ mL^−1^ and the test tubes were incubated at 25°C for 3 days under continuous illumination.

The activated culture was used as an inoculum for the following toxicity assessments.

For preliminary experiments, the exposure concentration of Ag or Pt nanoparticles of 5, 25 and 50 mg L^−1^ was chosen. During the main studies, the exposure concentration of Ag NPs was 1, 2, 3, 4 and 5 mg L^−1^ and of Pt NPs 5, 10, 15, 20 and 25 mg L^−1^. For the preparation of all exposure solutions 18 MΩ cm Milli-Q water (Millipore, USA) was used. Before the toxicity tests, exposure media were sonicated for 30 min. The nominal exposure concentration was confirmed via silver or platinum content determination in the initial solutions before algae exposure using inductively coupled plasma mass spectrometry (ICP MS). The differences between nominal and measured exposure concentrations were found to be below 4 %. Additionally, to test the presence of silver and platinum ions the initial solutions were filtered, ultracentrifuged and the amounts of silver/platinum in the suspensions were determined. The ion concentrations were undetectable, so it was assumed that algae were exposed to nanoparticles only.

Inhibition of the algal growth after 72 h of exposure was determined relative to controls by measuring optical density at 670 nm. Chlorophyll content was determined by spectrophotometric analysis (470, 645, 663 nm for total chlorophyll, chlorophyll *a* and chlorophyll *b*, respectively). The 72-h EC100 and EC50 values are the concentrations of the test substance which caused a 100 % or 50 % reduction in the rate growth relative to the control. Additionally, no observed effect concentration (NOEC) was determined for the investigated nanoparticles.

## Results and Discussion

**Fig. 1:**
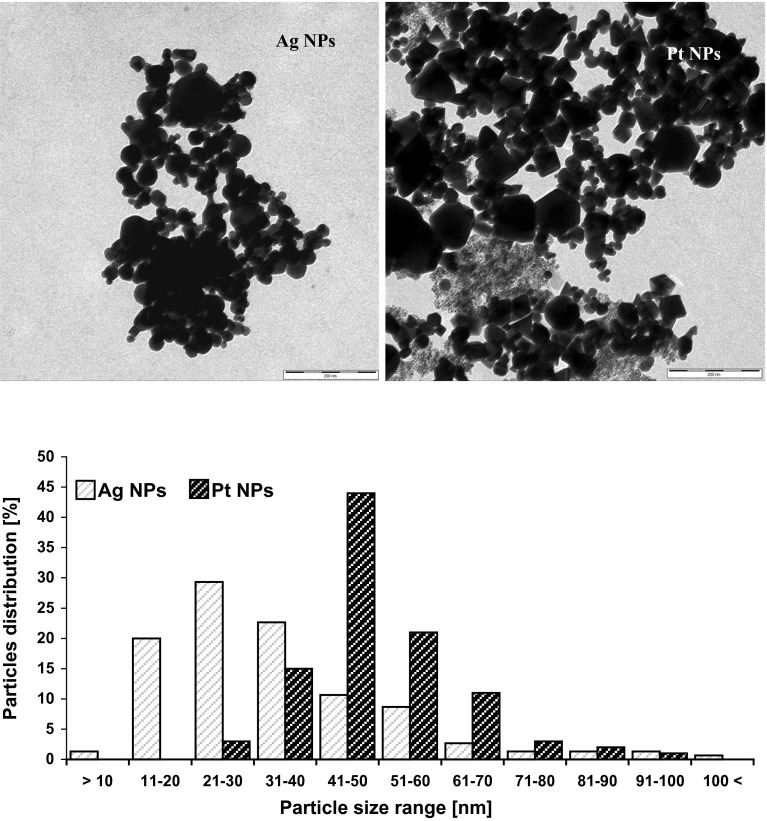
Transmission electron microscopy of silver and platinum nanoparticles (*scale bars*—200 nm) and particle size histograms of investigated NPs (1 mmol L^−1^ water solution). The sample pictures of Ag NP and Pt NP agglomerations show the variety of nanoparticle sizes and their shape

First, the silver and platinum nanoparticles were characterized using transmission electron microscopy (Fig. 1). The silver particles were spherical, while the platinum ones were irregular polyhedra. More than 70 % of silver nanoparticles were within the size range 10–40 nm, while nearly 80 % of platinum nanoparticles were within the range 30–60 nm. The average size of particles was 34 ± 18 and 51 ± 12 nm for Ag NPs and Pt NPs, respectively. A minor part of both nanoparticles was present in solution as agglomerates. The agglomerate size for silver and platinum was 200–600 and 250–1000 nm, respectively.

For the investigation of NP toxicity, the Algaltoxkit F test was performed. This is a 72-h assay based on the growth inhibition of the micro-alga *P. subcapitata*. This microbiotest strictly adheres to the protocols for regulatory testing with microalgae recommended by international organizations such as, the OECD and the ISO.

It is known that toxicity to *P. subcapitata* in a standard OECD test medium can be pH-dependent. During our experiments, the starting pH was 8.0 and this did not change during the incubation period (average pH 8.0 ± 0.1). Thus, there is likely no significant pH interference with our data.

**Fig. 2:**
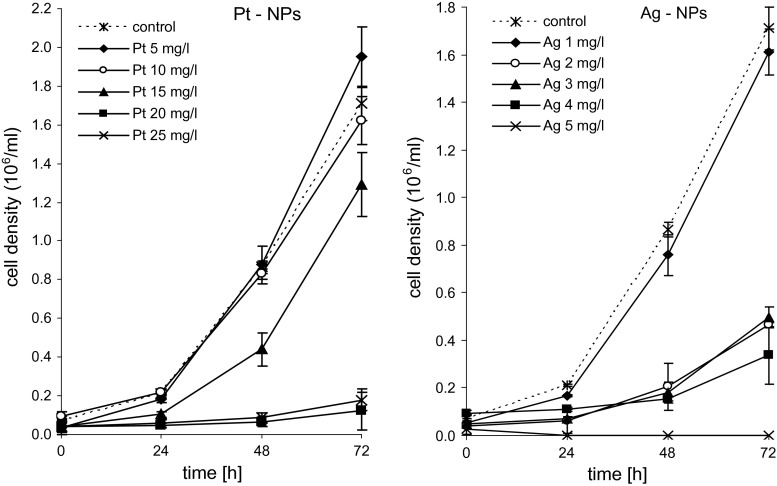
Growth kinetics of *Pseudokirchneriella subcapitata* in the presence of Ag and Pt nanoparticles. Values are means of three replicates ± standard deviation

The toxicity of the Ag and Pt nanoparticles was investigated within the concentration range of 1–50 mg L^−1^. Pilot experiments indicated that Ag NPs at ≥5 mg L^−1^ and Pt NPs at ≥25 mg L^−1^ fully inhibited the algal growth. To determine the EC100, EC50 and NOEC values, lower concentrations of the NPs were investigated. As illustrated by the growth curves in Fig. 2, Ag nanoparticles were highly toxic to *P. subcapitata* even at very low concentrations and the total inhibition of growth was observed at 5.0 mg L^−1^, all of which agreed with the pilot data. Compared to Ag nanoparticles, Pt-NPs were of remarkably lower toxicity to *P. subcapitata*. Substantial growth inhibition was only observed at ≥10 mg L^−1^, and for the total inhibition of the algal growth 22.2 mg L^−1^ Pt NPs was required. Table 1 provides the NOEC, EC50 and EC100 values for the two types of NPs. They are, respectively, ten-, twelve- and four-fold lower for silver than for platinum.

**Table 1:** The toxicity of silver and platinum nanoparticles to the alga *Pseudokirchneriella subcapitata* (72 h growth inhibition)

	Ag NPs (mg L^−1^)	Pt NPs (mg L^−1^)
NOEC	0.85	9.1
EC50	1.63	16.9
EC100	5.0	22.2

The toxicity of silver ions to aquatic invertebrates and algae has been studied extensively. The lowest recorded NOEC values varied between 0.001 μg L^−1^ (for daphnia) (Bielmyer et al. 2002) and 2 mg L^−1^ (for freshwater algae) (Ratte 1999). However, very little research has been done on the effects of Ag NPs on algal growth and photosynthesis (Miao et al. 2009; Navarro et al. 2008). Griffitt et al. (2009) investigated the toxicity of Ag NPs coated with sodium citrate to *P. subcapitata* (96-h test) and obtained an LC50 of 0.19 mg L^−1^, eight times lower than our result (1.63 mg L^−1^). This difference could be related to the particle size—in our studies the particles were fivefold larger. The coating of the particles could have played a role as well. In another study with the freshwater alga *Chlamydomonas reinhardtii*, Ag NPs appeared to be much more toxic than silver ions, indicating a specific NP effect (Navarro et al. 2008). The authors suggested that the high toxicity of Ag NPs was due to a high local concentration of Ag ions released from the NPs upon contact with the algal cell.

**Fig. 3:**
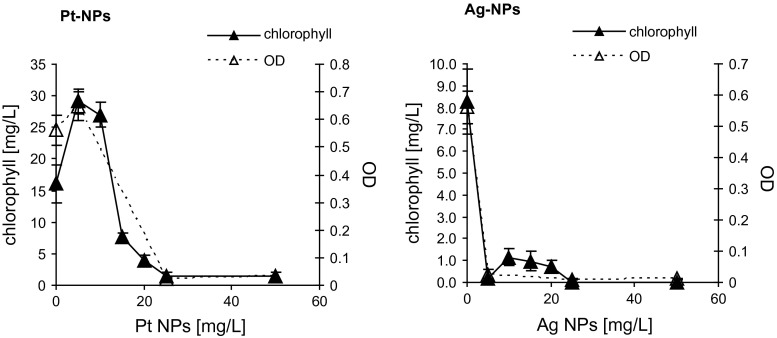
Effect of Ag or Pt nanoparticles on the optical density and chlorophyll content of *Pseudokirchneriella subcapitata* cultures

Photosynthesis is a well-known rate-limiting step in algal growth, and these effects will greatly influence the endpoint of the test. To check how the algal growth inhibition caused by the NPs is reflected in the chlorophyll content of the cultures, we determined total chlorophyll, or chlorophyll *a* and *b* separately, in 72-h cultures grown in the presence of various concentrations of Ag or Pt NPs. The results (Fig. 3) indicate that the total chlorophyll content of the culture is an excellent measure of the effect of NPs on algal growth, since the NP concentrations found to cause a 100 % drop of chlorophyll level were virtually identical to the EC100 values previously determined. An excellent correlation was found between the culture optical density, number of algal cells, and chlorophyll content (>90 %), indicating that the chlorophyll determination is a very good approach for the investigation of the toxicity of NPs to algae.

The purpose of this study was to elucidate the toxicity of silver and platinum nanoparticles toward the freshwater microalga *P. subcapitata* using a standardized algal growth inhibition test and the determination of photosynthetic pigments. Silver nanoparticles were more toxic than platinum NPs. We also found that the determination of chlorophyll can replace cell counting or other methods for biomass quantification.
